# Effect of ligustrazine nanoparticles on Th1/Th2 balance by TLR4/MyD88/NF-κB pathway in rats with postoperative peritoneal adhesion

**DOI:** 10.1186/s12893-021-01201-7

**Published:** 2021-04-26

**Authors:** Lili Yang, Ziyu Lian, Bin Zhang, Zhengjun Li, Li Zeng, Wenlin Li, Yaoyao Bian

**Affiliations:** 1 School of First Clinical Medicine, Nanjing University of Chinese Medicine, Nanjing, 210023 China; 2 Jingwen Library, Nanjing University of Chinese Medicine, Nanjing, 210023 China; 3 Jiangsu Provincial Engineering Center of TCM External Medication Researching and Industrializing, Nanjing, 210023 China; 4 Digestive Department, Ningbo Hospital of Traditional Chinese Medicine, Ningbo, 315012 China; 5 School of Management, University of St Andrews, St Andrews, KY16 9AJ UK; 6 School of Second Clinical Medicine, Nanjing University of Chinese Medicine, 138 Xianlin Rd., Nanjing, 210023 China; 7 School of Nursing, Nanjing University of Chinese Medicine, 138 Xianlin Rd., Nanjing, 210023 China; 8 TCM Nursing Intervention Laboratory of Chronic Disease Key Laboratory, Nanjing, 210023 China

**Keywords:** Postoperative peritoneal adhesion, Ligustrazine nanoparticle, Th1/Th2 balance, TLR4/MyD88/NF-κB pathway

## Abstract

**Background:**

Postoperative peritoneal adhesion (PPA) is regarded as fibrous bands connecting both injured abdominal wall and organs or adjacent tissues. It is associated with T helper (Th)1 and Th2 differentiation. However, the critical role of the immunopathogenesis of adhesion formation was precisely unknown. The aim of this study was to investigate the effect of a new agent polylactic acid (PLA) nanoparticles loaded with ligustrazine, that is, ligustrazine nanoparticles (LN) on PPA and identify the potential mechanism.

**Methods:**

Twenty-four Sprague–Dawley rats were randomly divided into the sham, model, LN, and sodium hyaluronate (SH) groups. The structure of LN, including entrapment efficiency (EE) and loading capacity (LC), and in vitro drug release were calculated. Adhesions were scored and the Masson's trichrome staining was used to determine the collagen deposition. The expressions of TLR4, MyD88, and NF-κB were measured by qRT-PCR, immunohistochemistry, and western blot assay. Moreover, Th1-related cytokines (IFN-γ, IL-12), Th2-related cytokines (IL-4, IL-6) in the cecum tissue and serum were conducted by ELISA.

**Results:**

LN had good EE, LC, and control-release delivery characters with fairly uniform diameter and spherical morphology. It could effectively prevent adhesion formation after surgery. Besides, it could reduce collagen fibers accumulation, downregulate the expression levels of TLR4, MyD88, and NF-κB, and maintain Th1/Th2 balance.

**Conclusions:**

Ligustrazine nanoparticles had effective effects on Th1/Th2 balance by regulating TLR4/MyD88/NF-κB pathway in PPA rats. It may be served as a promising therapy on postoperative adhesion formation.

## Background

Postoperative peritoneal adhesion (PPA) is regarded as fibrous bands connecting both injured abdominal wall and organs or adjacent tissues. It remains one of the most challenging issues in surgery fields around the world. Approximately 50–85% of patients who underwent abdominal surgery suffered from various adhesion-associated problems including abdominal pain, female infertility, and intestinal obstruction [1, 2]. In the USA, about 117 per 100,000 people re-hospitalized due to the above complications, and the direct cost spent on adhesive complications reached up to $1.3 billion [3, 4]. It brought out a tremendous burden on public health care.

The formation of adhesion is a multistep process that is related to oxidative stress [5], inflammatory response, and the fibrinolytic system [6]. However, the critical role of immunopathogenesis was precisely unknown. Few studies suggested that immune progress controlled by T cells played prominent roles in adhesion formation [7, 8]. T cells can be divided into two subpopulations, that is T helper (Th)1 and Th2 [9]. Mounting evidence indicated that Th1/Th2 ratio would shift due to the major or minor abdominal surgery [10, 11]. Th1 cells are the effector cells in mediating adhesion formation [12]. And a significant increase of Th2 cells was found in the adhesion area of a rodent experiment [8]. Previously, we reported that the exact roles of the TLR4/MyD88/NF-κB signaling pathway in the pathogenesis of PPA based on the microarray analysis [13]. Herein, it might be a beneficial way to find a new approach to regulate Th1/Th2 balance through TLR4/MyD88/NF-κB regulatory chain.

We previously reported that ligustrazine, a kind of bioactive component derived from the root of herbal *Ligusticum Chuanxiong hort* (*Umbelliferae*) had positive effects on adhesion formation in *vivo* and in vitro [14, 15]. However, the disadvantages of ligustrazine on injured sites i.e. rapid absorption and metabolism, short half-life, and uneven distribution limited its application in biomedical fields. The effects of ligustrazine on pre-clinical studies are far from satisfactory. A recent study indicated that ligustrazine-polylactic acid (PLA) sustained-release microspheres might be a promising agent due to the characteristics of stable and sustained release effects on bioactive ingredients [16]. As PLA is emerging as an ideal drug carrier for its favorable biocompatibility and biodegradable [17]. To enhance the specificity of ligustrazine delivery to injured tissues, the new agent PLA nanoparticles loaded with ligustrazine, that is, ligustrazine nanoparticles (LN), were used in our study. We aimed to explore the exact effects of LN on PPA and identify the possible mechanism.

## Methods

### Preparation and microstructure of LN

Ligustrazine (C_8_H_12_N_2_, purity ≥ 98%) was provided by Tokyo Chemical Industry (Japan). The LN was prepared as our previous studies reported [18, 19]. An appropriate amount of ligustrazine and PLA (1:4) were taken and dissolved in the acetone solution. The mass concentration of PLA in the acetone solution should be 20 g/L. This solution was called the oil phase. 0.25% poloxamer solution with four times the volume of the oil phase was used as the water phase. Then, the oil phase was poured rapidly into the water phase at a high agitation speed at 30℃, and the two phases were kept stir for 70 min until the acetone was evaporated. Finally, 1 mg/mL ligustrazine nanoparticles were got. One-drop sample of the above LN was dripped onto the copper grid with a thin carbon film. After the drop was air-dried, phosphotungstic acetate solution was applied to stain for 5 min. The microstructure of LN was visualized under the high resolution transmission electron microscope (HRTEM, Japan).

### Determination of entrapment efficiency (EE) and loading capacity (LC)

EE and LC are very important indicators to measure the properties of nanoparticles. LN was centrifuged at 25,000 r/min for 60 min, and the supernatant was harvested to measure EE and LC. About 0.2 mL supernatant mixed with 4.8 mL methanol were used to calculate the contents of free ligustrazine in the supernatant under the high-performance liquid chromatography (HPLC), which was regarded as W_f_. And 0.2 mL LN mixed with 0.5 mL N, N-dimethylformamide, and 4.3 mL methanol were applied to calculate the total amount of LN under HPLC, which was named as W_t_. EE and LC were calculated as follows[20]:

$${\text{EE(\% ) = [(W}}_{{\text{t}}} - {\text{W}}_{{\text{f}}} {\text{)/W}}_{{\text{t}}} {] }$$

$${\text{LC(\% ) = [(W}}_{{\text{t}}} {\text{ - W}}_{{\text{f}}} {\text{) / W}}_{{\text{n}}} {] }$$

Whereas, W_n_ was the weight of LN after lyophilization.

### In vitro drug release

Because ligustrazine was a lipid-soluble drug, phosphate buffer was used as a release medium. The phosphate buffer was prepared as follows: 1.36 g potassium dihydrogen phosphate mixed with 79 mL 0.1 mol/L sodium hydroxide solution, and diluted in deionized water up to 200 mL. Then the mixed solution was re-suspended with 0.25% poloxamer. 1 mL LN was added in a membrane dialysis bag in 30 mL above release medium at PH = 7.4 at room temperature with stirring constantly. During this time, 1 mL dialysis buffer was taken out of the bag every five minutes, and the same volume release medium at the same temperature was added. The concentration of the released drug was calculated by HPLC.

### Animal model preparation and group assignment

Twenty-four healthy male adult Sprague–Dawley rats (weighting 220 ± 20 g) were provided by Experiment Animal Center of Nanjing University of Chinese Medicine. The rats were all housed in a controlled condition with temperature (18–25℃) and relative humidity (65–70%) on a reverse 12 h light/dark circle. After acclimating for one week, the rats were randomly divided into 4 groups of 6 rats each, that is, sham, model, LN, and sodium hyaluronate (SH) groups. This study was approved by the Ethics Committee of Nanjing University of Chinese Medicine (No. ACU171112).

The method of model preparation is a patent of Pro. Li Zeng [21]. It is also an effective way to prepare PPA models [22, 23]. In brief, all rats were fasted for 12 h but allowed to drink water before the experiment. All surgical procedures were conducted under aseptic conditions. After anesthetized with 1–1.5% isoflurane, rats were skin prepared and maintained at supine position. A 1.5–2 cm incision was made on the midline of the lower abdomen. The cecum was rubbed smoothly and repeatedly by a needle file until petechiae appeared on the serosa layer. Then the injured cecum was replaced into the abdominal cavity and sewed layer by layer. The cecum of rats in the sham group was only exposed in the air for 5 min without rubbing. In the LN and SH groups, 5 ml/kg LN and 0.5 ml/kg SH gel (Bausch & lomb) was sprayed on the injured cecum and surrounding area before closing the abdominal cavity, respectively. All rats were anesthetized with 1–1.5% isoflurane on the 7^th^ day after the operation and an inverted U-incision was used to open the abdominal cavity. The blood samples and cecum tissues, especially the adhesive sites, were collected for the following analysis. After hemostasis was done completely, the abdominal wall was closed. All rats were then euthanized by sodium pentobarbital (200 mg/kg).

### Adhesion grading and evaluation

The degree of adhesion was determined using a five-stage adhesion score system [13, 24, 25] by two independent investigators in a blinded way. This scoring system included five stages adhesion scores ranging from 0 to 4. Grade 0 represents no adhesion areas; grade 1 represents 0–25% zones with thin, avascular, and transparent adhesion, indicating a milder inflammatory response; grade 2 represents 25–75% zones with thick, avascular, and opaque adhesion, meaning a mild inflammatory reaction; grade 3 represents 50–75% thick, capillaries, opaque adhesion, and sharp dissection required, indicating a moderate response; grade 4 represents 75–100% thick, opaque adhesion with large vessels, and sharp dissection required, meaning a severe reaction.

### Masson’s trichrome (MT) staining

A portion of the cecum specimens was placed in 10% formaldehyde for 24 h and dehydrated using graded ethanol. Then, the tissues were embedded in paraffin, cut into 4 μm thick sections. After the sections were heated and dewaxed, they were stained with MT kit (Leagene Biotechnology, Beijing) according to routine protocols. Different fields were chosen randomly by microscope (DM2500; Leica, Germany) to evaluate the inflammation.

### Enzyme-linked immunosorbent assay (ELISA)

The cecum tissue was stored in − 80℃ for the following analysis. The blood samples were centrifuged at 3000 rpm for 20 min. Afterward, the supernatant was collected and stored in 4℃ for the subsequent analysis. The concentrations of IFN-γ, IL-12, IL-4, and IL-6 in the cecum tissue and serum were determined by ELISA kit (JinYiBai, Nanjing) according to the instructions. The optical density values with a wavelength of 450 nm were determined by the enzymatic analyzer (Tecan F50, Swiss).

### Western blotting (WB)

Protein from cecum tissues was extracted by a lysis buffer. The protein concentration was measured by BCA kit (Beyotime, Shanghai). The protein was separated by sodium dodecyl sulfate–polyacrylamide gel electrophoresis (Bio-Rad, USA). After transferred onto the polyvinylidene fluoride membranes (Millipore, USA), the membranes were blocked in the blocking buffer for 1 h at 37℃, and incubated with the primary antibodies at 4℃ overnight. The primary antibodies included anti-TLR4 (1:200 dilution), MyD88 (1:200 dilution), NF-κB (1:200 dilution), β-actin (1:2000 dilution) were all provided from Santa Cruz Biotechnology (USA). Then, the membranes were rinsed and incubated with the second antibodies for 80 min at 37℃. Finally, the protein expression was imaged by the Chemiluminescence Imaging System (Bio-Rad, USA), and bands were measured by ImageLab Software.

### Immunohistochemistry

After the cecum tissues were fixed in 10% formaldehyde for 24 h and embedded in paraffin wax, the tissues were cut into sections. These sections were developed using Diaminobenzidine tetrahydrochloride (DAB) kits (CWBIO, Beijing). The sections of 4-μm thickness were incubated with NF-κB (1:200 dilution) overnight at 4℃. The second antibody and color were all conducted according to the DAB kits’ instruction. Views were randomly visualized under a microscope (DM2500; Leica, Germany).

### Quantitative reverse transcription PCR (qRT-PCR)

Total RNA from the cecum samples was extracted by Trizol reagent (Invitrogen, USA). Nanodrop 2000 was applied to determine the RNA concentration. Based on the manufacturer’s instruction of RevertAid*™*, cDNA was synthesized in a reverse transcript manner. According to the standard protocol, the qRT-PCR was performed using SYBR*™* Green Master Mix (Thermo, USA). The relative changes of mRNA expression were analyzed by the comparative Ct method as previously described[26]. The primers were presented in Table 1.

**Table 1:** the primers used for qRT-PCR

Gene	Primers sequence
TLR4	F 5′-3′ TGAATCCCTGCATAGAGGTA
	R 5′-3′GACCGTTCTGTCATGGAAGG
MyD88	F 5′-3′GTAGCCAGCCTCTGAAAC
	R 5′-3′AGCCAGGATGATGTCTAC
NF-κB	F 5′-3′AGTTGAGGGGACTTTCCCAGGC
	R 5′-3′GATTCGAGTATTAGTTCATGGA
β-actin	F 5′-3′TCCTCACTGAGGCCCCGC
	R 5′-3′CTGCCCCATGCCATTCTC

### Statistical analysis

All experiments were performed in triplicate. All data were analyzed using SPSS 22.0 and were presented as mean ± standard deviation. Multiple comparisons were performed using one-way analysis of variance followed by LSD test. The Kruskal–Wallis test was used for non-normally distributed continuous data. A level of *P* < 0.05 was regarded as statistically significant.

## Results

### Physicochemical characterization of LN

**Fig. 1:**
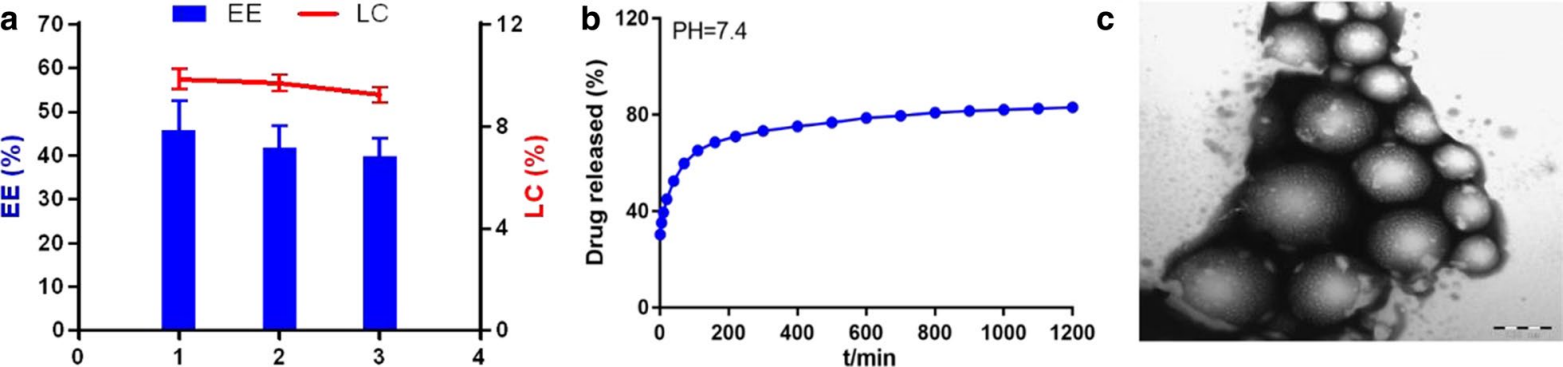
Physicochemical characterization of ligustrazine nanoparticles (LN). **a** Entrapment efficiency (EE) and loading capacity (LC) of LN. **b** Cumulative release curve of LN at PH = 7.4. **c** Scanning electron microscope image of LN (×200 nm)

The drug delivery efficiency of PLA loaded with ligustrazine was determined using two EE and LC indicators, as presented in Fig. 1a. It suggested that LN had good entrapment efficiency and loading capacity. Kinetic study of drug release at PH = 7.4 value was shown in Fig. 1b. And the microstructure of LN was a spherical shape with a smooth surface (Fig. 1c). The diameter distribution of nanoparticle is about 200 nm. The results indicated that PLA loaded with ligustrazine nanoparticles had good biocompatibility and target delivery, which might be a promising agent for preventing adhesion formation.

### LN lessened the formation of PPA in rats

**Fig. 2:**
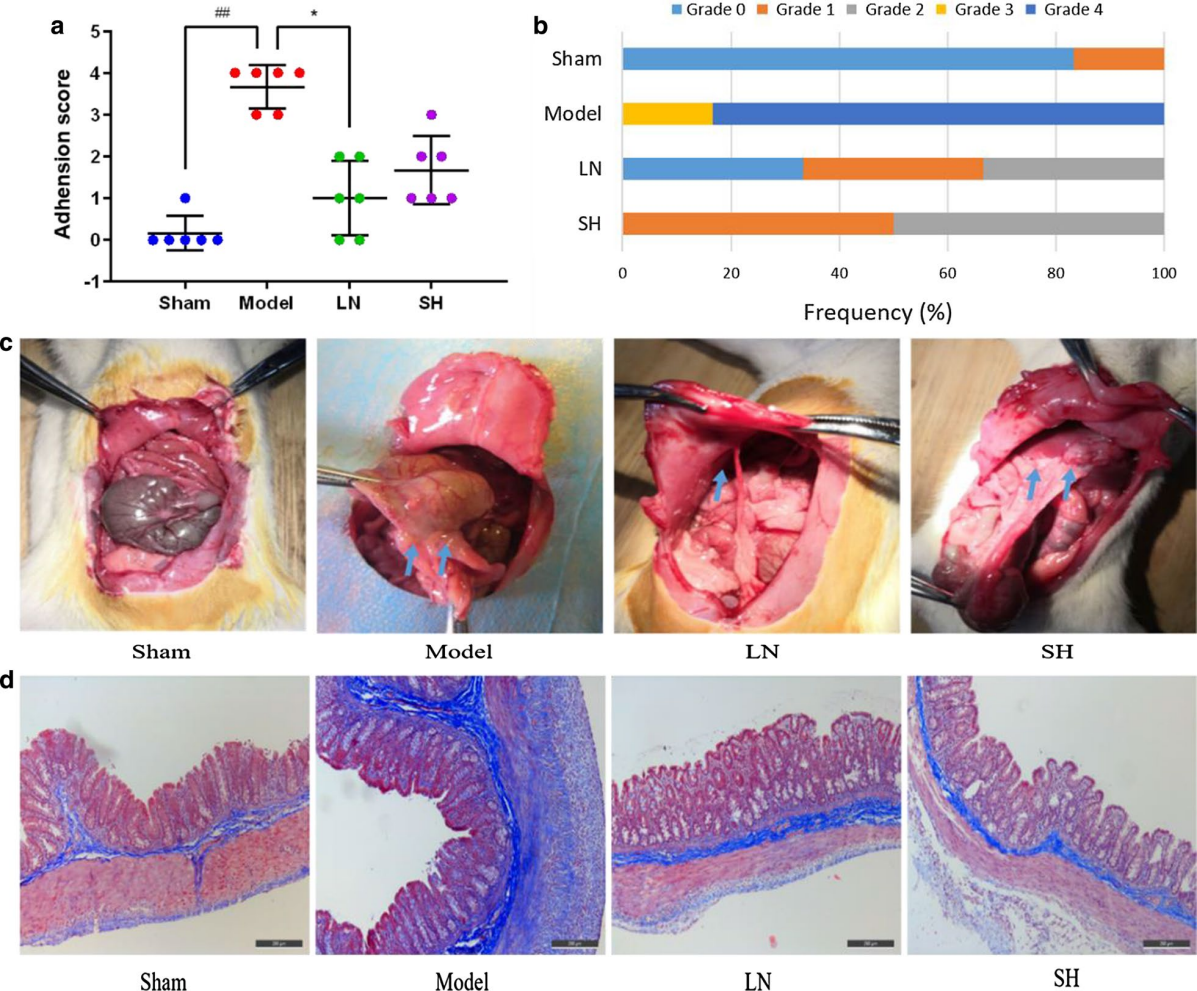
Effect of LN on reducing adhesion formation. **a** Adhesion score of four groups. Compared with the sham group, ^#^*p* < 0.05, ^##^*p* < 0.01. Compared with the model group, *p < 0.05, **p < 0.01. **b** Frequency of different grades in four groups. **c** Representative images of adhesion formation of different groups. **d** Representative images of Masson’s trichrome staining of different groups (100×)

We found that no rats died after surgery and no significant difference in weight among the four groups. Among the twenty-four rats, two rats in the model group and one in the SH group might have intestinal obstruction or necrosis due to the cecum appeared dark black. The adhesion scores of all groups were summarized in Fig. 2a. Compared with the sham group, the model group exhibited a severe peritoneal adhesion with a higher adhesion score (*p* = 0.00). The adhesion score of the LN group was significantly lower than that of the model groups (*p* = 0.03). The frequency of different grades and representative images in four groups were presented in Fig. 2b and Fig. 2c, respectively. The results suggested that LN could significantly lessen adhesion formation.

### LN suppressed collagen deposition in PPA rats

Collagen deposition was one of the important pathological processes followed by the inflammatory reaction during the adhesion formation. Masson staining was used to determine the density of collagen fibers deposition among different groups, as shown in Fig. 2d. Compared with the sham group, there were massive inflammatory cells and collagen fibers in the model group. In comparison with the model group, the fibrin thickness significantly decreased in the LN group. The findings indicated that LN could suppress collagen fibers accumulation.

### LN activated the TLR4/MyD88/NF-κB pathway

**Fig. 3:**
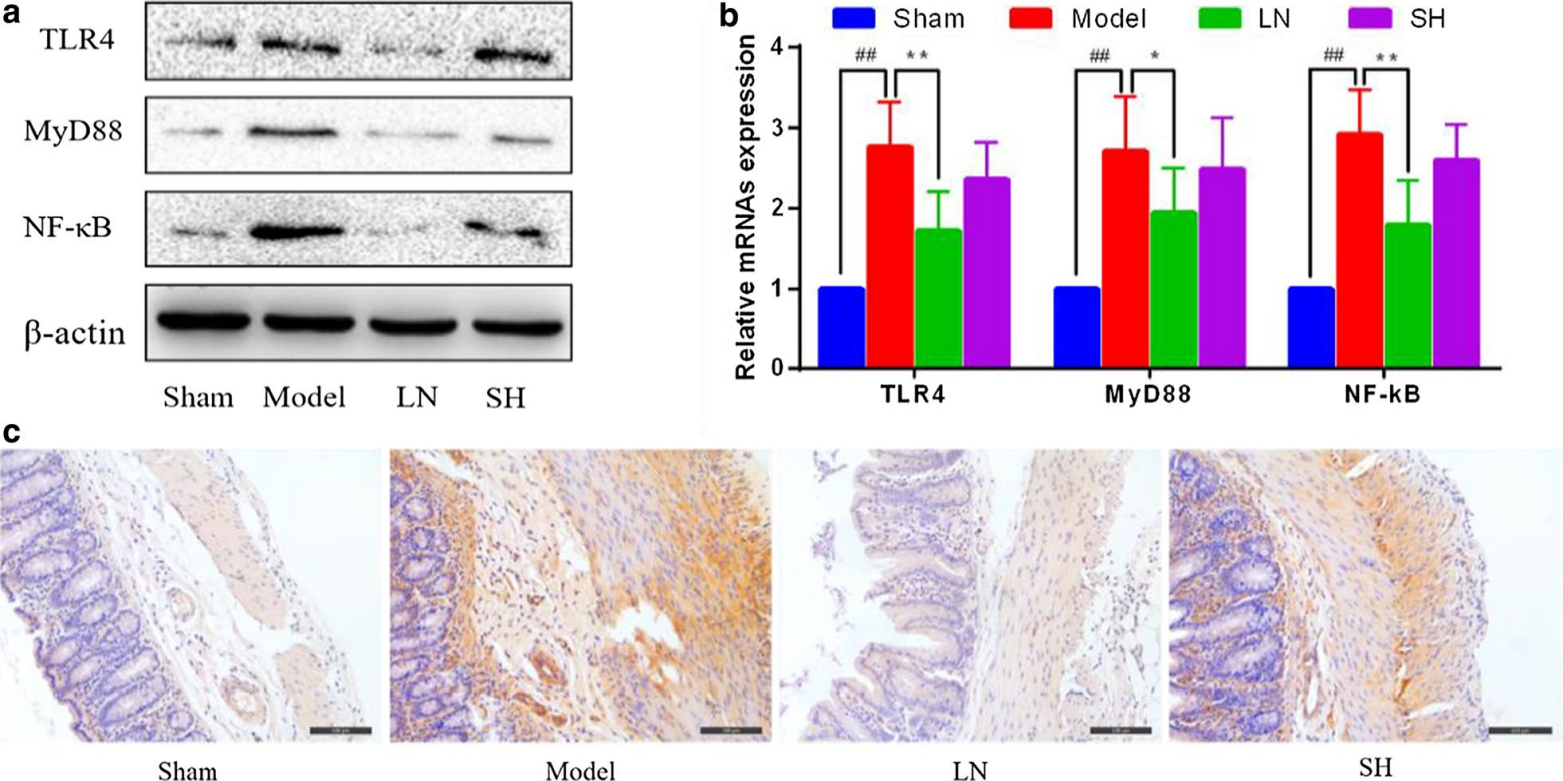
Effect of LN on activating the TLR4/MyD88/NF-κB pathway. **a** The expression levels of TLR4, MyD88, and NF-κB by western blot. **b** The mRNAs levels of TLR4, MyD88, and NF-κB by quantitative reverse transcription PCR (qRT-PCR) assay. Compared with the sham group, ^#^*p* < 0.05, ^##^*p* < 0.01. Compared with the model group, ^*^p < 0.05, ^**^p < 0.01. **c** Representative images of immunohistochemical staining of NF-κB in different groups (200×)

To explore the critical effects of LN on the TLR4/MyD88/NF-κB pathway, WB and qRT-PCR were applied. In comparison with the sham group, the protein levels of TLR4, MyD88 and NF-κB were significantly upregulated in the model group. After LN treatment, TLR4, MyD88, and NF-κB levels were downregulated, as presented in Fig. 3a. The results were in line with those of the qRT-PCR (Fig. 3b). Compared with the sham group, the TLR4, MyD88, and NF-κB expressions in the model group increased significantly (TLR4 *p* = 0.000000; MyD88 *p* = 0.000027; NF-κB *p* = 0.000000). In comparison with the model group, the levels of above mRNAs in the LN group decreased markedly (TLR4 *p* = 0.000490; MyD88 *p* = 0.023910; NF-κB *p* = 0.000354). Besides, the immunohistochemical analysis of NF-κB in four groups was shown in Fig. 3c. The findings revealed that the LN could lessen adhesion formation by activating the TLR4/MyD88/NF-κB pathway.

### LN improved the Th1/Th2 balance in PPA rats

**Fig. 4:**
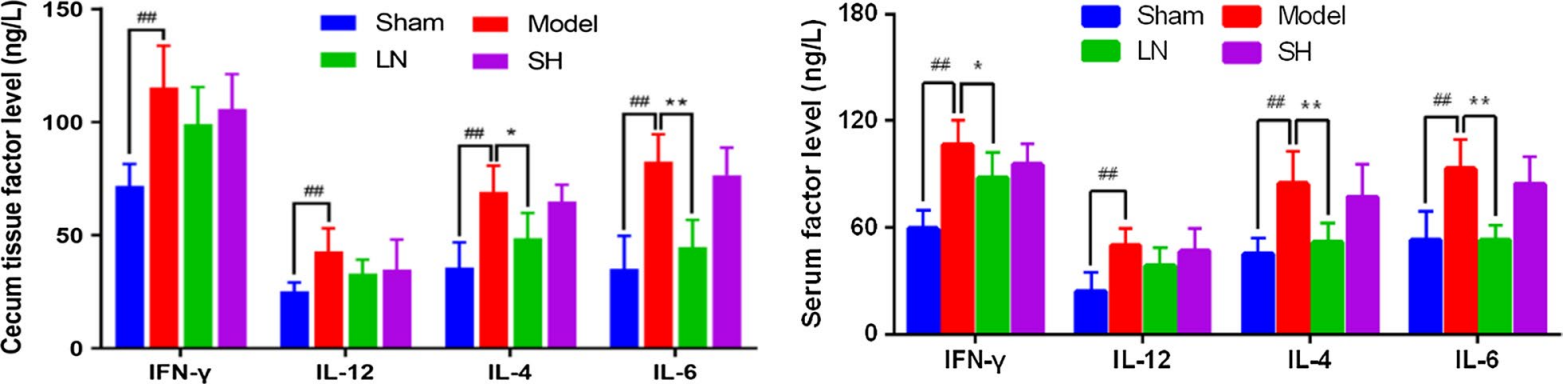
Effect of LN on improving the Th1/Th2 balance. The levels of Th1-related cytokines (IFN-γ, IL-12) and Th2-related cytokines (IL-4, IL-6) in the cecum tissues and serum by enzyme-linked immunosorbent assay (ELISA) assay. Compared with the sham group, ^#^*p* < 0.05, ^##^*p* < 0.01. Compared with the model group, *p < 0.05, **p < 0.01

To further determine LN on the downstream of TLR4/MyD88/NF-κB pathway, the levels of Th1-related cytokines (IFN-γ, IL-12) and Th2-related cytokines (IL-4, IL-6) in serum and cecum tissues were measured by ELISA, respectively. Compared with the sham group, both Th1- and Th2-related cytokines levels in cecum tissues and serum elevated in the model group (IFN-γ in cecum *p* = 0.000085; IL-12 in cecum *p* = 0.003065; IL-4 in cecum *p* = 0.000021; IL-6 in cecum *p* = 0.000003; IFN-γ in serum *p* = 0.000003; IL-12 in serum *p* = 0.000641; IL-4 in serum *p* = 0.000165; IL-6 in serum *p* = 0.000116). After LN intervention, four cytokines decreased. But there were no significant differences of IFN-γ and IL-12 in cecum tissues and IL-12 in serum. The IFN-γ levels in serum showed a significant decreased after LN treatment (*p* = 0.019614). Moreover, IL-4 and IL-6 levels in cecum tissues and serum downregulated significantly after LN intervention (IL-4 in cecum *p* = 0.002997; IL-6 in cecum *p* = 0.000051; IL-4 in serum *p* = 0.001003; IL-6 in serum *p* = 0.000113), as shown in Fig. 4. The results demonstrated that LN could maintain Th1/Th2 balance by TLR4/MyD88/NF-κB pathway in PPA rats.

## Discussion

Ligustrazine is an alkaloid monomer of the plants *Chuangxiong* which is widely used in a variety of diseases, such as cardiovascular diseases [27]. In recent years, various evidence demonstrated that ligustrazine had anti-oxidant and immunity functions [28]. Previously, we had reported that ligustrazine could suppress peritoneal fibrosis so as to prevent adhesion formation [14, 15]. In order to improve its bioavailability, we used nanotechnology in the current study. PLA was chosen as the ideal nanoparticle carrier due to its lipophilic, biodegradable, and biocompatible behaviors on cancer and other treatment [29, 30]. PLA loaded with ligustrazine was applied in our study, which filled up the short-term metabolism and rapid absorption of ligustrazine in rodent models effectively. We found that LN had stable and sustained release behavior. LN with fairly uniform diameter and spherical morphology is appropriate and effective for ligustrazine targeting-delivery on injured sites.

Postsurgical adhesion formation following cecal abrasion is triggered by the inflammatory reaction, during which various macrophages and neutrophils are activated and proliferated to the injured sites. These cells are regarded as important leukocytes in abdominal immunity [31]. The activated T cells move to the abdominal cavity, coordinate chemokine production and leukocyte transportation, and home to the adhesion sites [32]. T cells and their secreted cytokines and chemokines play pivotal roles in the initiation and development of adhesion formation [33]. It was reported that the shifting of Th1 and Th2 contributed to the severity of adhesion [12, 34]. IFN-γ was selectively recruited in the pathogenic tissue response [7]. IL-4 as an important Th2 cytokine inhibited the development of Th1 cells. It was reported to involve in the pathogenesis of wound healing [35]. Up-regulated expression of IL-6 is found in PPA models [1, 36]. IL-6 can differentiate naïve CD4^+^ αβT cells into Th17 cells which contributed to the pathophysiologic process of immune disorders [37]. IL-12 is produced by activated macrophages. It acts an important role in the activation and proliferation of T cells [38].

In our previous study, we found that the critical regulatory pathway of TLR4/MyD88/NF-κB according to bioinformatics analysis. And we verified several hub genes including IL-6 on PPA models preliminarily. In this study, we found that the Th1-released IFN-γ and IL-12 levels, as well as Th2-released IL-4 and IL-6 levels, increased in the model group when compared with the sham group. After LN intervention, the expression levels of Th1 (IFN-γ) in serum, and Th2 (IL-4 and IL-6) in cecum tissue and serum showed significant differences. All evidence suggested that LN could activate TLR4/MyD88/NF-κB pathway, regulate the downstream cytokines expression, induce Th1/Th2 differentiation and maintain Th1/Th2 balance to prevent adhesion formation. IL-4 produced by Th2 cells inhibits the production of IL-12 and IFN-γ secreted by Th1 cells [39]. IL-6 can not only induce IL-4 expression to promote Th2 differentiation but also suppress IFN-γ production to inhibit Th1 differentiation [40]. Due to the dual roles of IL-4 and IL-6, Th2 cells might play a more important role in the T-cell mediated immunity. One major limitation of our study is that the study is based on the rodent model, and the effect of LN on human adhesion formation needs further investigation.

## Conclusion

In conclusion, the crucial finding of our study was that LN could markedly prevent postoperative adhesion formation. The underlying mechanism might be related to the activation of TLR4/MyD88/NF-κB pathway and the balance of Th1/Th2 differentiation. LN can be regarded as a promising agent against postoperative peritoneal adhesion.

## Data Availability

The data are available from the corresponding authors on reasonable request.

## Abbreviations

PPA: Postoperative peritoneal adhesion
Th1: T helper 1
Th2: T helper 2
PLA: Polylactic acid
LN: Ligustrazine nanoparticles
SH: Sodium hyaluronate
EE: Entrapment efficiency
LC: Loading capacity
HPLC: High performance liquid chromatography
DAB: Diaminobenzidine tetrahydrochloride
qRT-PCR: Quantitative reverse transcription PCR
MT: Masson’s trichrome
ELISA: Enzyme-linked immunosorbent assay
WB: Western blotting
